# Refractory Ventricular Tachycardia From Coronary Vasospasm During Pregnancy

**DOI:** 10.31486/toj.18.0046

**Published:** 2019

**Authors:** Kevin Ergle, Michael Bernard

**Affiliations:** Department of Cardiology, Ochsner Clinic Foundation, New Orleans, LA

**Keywords:** *Coronary vasospasm*, *pregnancy complications–cardiovascular*, *tachycardia–ventricular*

## Abstract

**Background:** Coronary vasospasm leading to variant angina is uncommon, and the condition is rare in pregnant patients. Many physiologic changes occur during pregnancy, but how these changes affect the spasticity of coronary arteries in patients predisposed to vasospasm is unknown. Vasospasm causing unstable arrhythmia from multiple foci can be difficult to treat.

**Case Report:** A 22-year-old gravida 1 para 0 female at 17 weeks’ gestation with twins presented with chest pain refractory to sublingual nitroglycerin, ST segment elevation on electrocardiogram, and subsequent ventricular tachycardia requiring a shock by her implantable cardioverter defibrillator (ICD). The patient had a history of coronary vasospasm with ventricular arrhythmia that required placement of the ICD 5 years prior. Because of refractory symptoms, she required prolonged admission in the intensive care unit with high-dose intravenous nitroglycerin, calcium channel blockers, benzodiazepines, beta blockers, chemical sympathectomy, and intubation and sedation. Despite these measures, the patient continued to have vasospasm and ventricular tachycardia, so cesarean delivery and tubal ligation were performed. After delivery, she was rapidly weaned from all invasive treatment modalities and was discharged on oral nitrates and calcium channel blockers.

**Conclusion:** To our knowledge, this case is the first report of severe drug-refractory vasospastic angina triggered by pregnancy. The hormonal and nervous system changes that occur during pregnancy appear to be a trigger for vasospasm, further highlighted by the quick resolution of the patient's symptoms postdelivery. A multidisciplinary approach for treatment of both mother and baby was necessary. Our case provides a cautionary tale that patients with refractory vasospastic angina may want to pursue definitive contraception.

## INTRODUCTION

In 1959, Prinzmetal and colleagues described a “variant type of angina” that occurred frequently at rest or during normal activity and was not worsened by effort as classic Heberden angina pectoris is.^1^ This variant form of angina—a type of vasospastic angina in which spasm of an epicardial coronary artery leads to myocardial ischemia with representative ST segment changes—is relatively uncommon, with a higher prevalence in eastern countries. Among patients with vasospastic angina, the majority will have single-vessel vasospasm, although data from a multicenter registry of the Japanese Coronary Spasm Association suggest that up to 32% of patients can have multivessel territories.^2^ Only approximately 2.4% of patients with coronary vasospasm will have out-of-hospital cardiac arrest.^3^

Smoking is a major preventable risk factor, with male sex, magnesium deficiency, alcohol use, physical and mental stress, and autonomic nervous system agents all having an association with vasospastic angina.^4^ Treatment modalities include nitrates, calcium channel blockers, nicorandil (not available in the United States), and concomitant percutaneous coronary intervention for organic stenoses.^5^ Implantation of an automated implantable cardioverter defibrillator (ICD) is reasonable for patients with prior ventricular arrhythmia or cardiac arrest.^5^

The incidence of ischemic heart disease during pregnancy is low, occurring in only 1 in 10,000 patients.^6^ A review of 103 patients with acute myocardial infarction in pregnancy found only 2 cases attributed to coronary spasm.^7^ The effects that pregnancy may have on someone with a history of vasospastic angina, particularly patients with intractable vasospastic angina contributing to unstable cardiac arrhythmia, are unknown.

## CASE REPORT

A 22-year-old primigravid female at 17 weeks’ gestation presented with chest pain refractory to sublingual nitroglycerin and an appropriate shock by her ICD. She had a history of anxiety, depression, and angiography-documented coronary vasospasm in multiple coronary territories. Prior cardiac arrest because of ventricular tachycardia that degenerated to ventricular fibrillation led to implantation of the ICD 5 years prior. Family history was negative for sudden cardiac death.

The patient was chronically maintained on verapamil 120 mg 3 times daily, isosorbide mononitrate 60 mg daily, and magnesium supplementation. She had a history of 7 ICD shocks, the last almost 4 years prior to this presentation. Starting at 9 weeks’ gestation of her pregnancy, she had had several admissions to outside facilities for angina requiring intravenous (IV) nitroglycerin administration, but those events were self-limited and no arrhythmia was noted.

**Figure 1.**
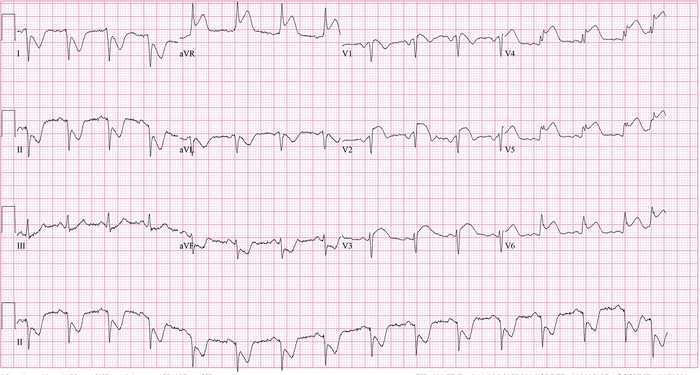
**Admission electrocardiogram shows anteroseptal and lateral ST segment elevation.**

Electrocardiogram (ECG) on admission showed antero-septal ST segment elevation (Figure 1). Fetal ultrasound showed twin pregnancy with only one surviving fetus on admission. Echocardiogram showed mildly depressed left ventricular function with septal hypokinesis. The patient was admitted to the intensive care unit, and IV nitroglycerin was started. ECG changes and the patient's chest pain resolved. The night after admission, however, she felt crushing chest pain and had 6 appropriate shocks by her ICD for ventricular fibrillation. She was intubated, sedated on propofol and fentanyl continuous IV infusions, and continued on IV nitroglycerin to 500 mcg/min and verapamil 120 mg 3 times daily.

**Figure 2.**
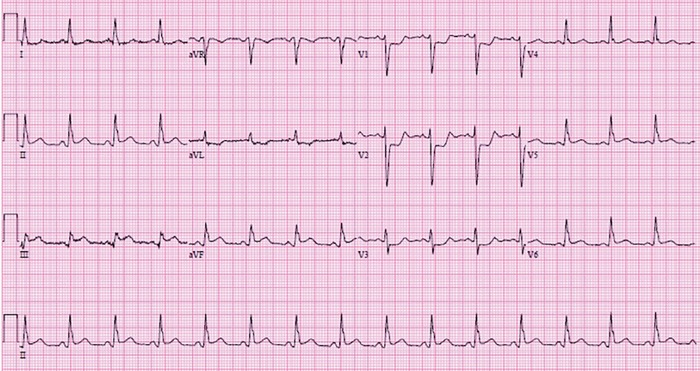
**Inpatient electrocardiogram shows inferior ST segment elevation.**

**Figure 3.**
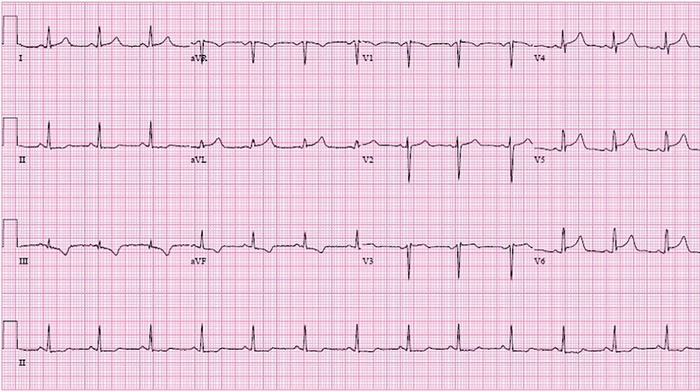
**Inpatient electrocardiogram shows lateral ST segment elevation.**

Multidisciplinary discussion involving electrophysiology, maternal-fetal medicine, cardiology, and interventional cardiology determined that given the patient's history, vasospastic angina was likely the cause of the arrhythmia, and alternative diagnoses such as spontaneous coronary artery dissection were less likely. Subsequent ECGs during her hospitalization showed ST segment elevation in different territories, further solidifying the diagnosis (Figures 2 and 3).

Despite these initial measures, the patient continued to have angina with refractory ventricular tachycardia and ventricular fibrillation. In addition to high-dose intravenous nitroglycerin 500 mcg/min, dihydropyridine and nondihydropyridine calcium channel blockers (nifedipine 90 mg daily and verapamil 240 mg 3 times daily), and strict electrolyte control, the patient was treated with benzodiazepines (scheduled diazepam 7 mg 3 times daily with lorazepam as needed), a beta blocker (metoprolol tartrate 25 mg 3 times daily), and sedation with intubation to attempt to control her coronary vasospasm. Psychiatry was consulted to help with control of her anxiety. Despite these treatments, she continued to have refractory tachyarrhythmias, leading to multiple appropriate shocks by her ICD. After discussion with thoracic surgery and anesthesiology, chemical sympathectomy was performed with ropivacaine 4 mg/hr via a stellate ganglion catheter. This treatment initially improved the patient's angina; however, at 24-weeks’ gestation she again developed refractory ventricular tachycardia. In total, she had more than 20 shocks by her ICD by this point in her pregnancy.

Discussion with the patient, her family, maternal-fetal medicine, obstetrics, critical care, and cardiology concluded that the best option for the patient and the baby was to proceed with cesarean section and delivery of the baby, which occurred at 24 weeks, 6 days. With consent of the patient and family, permanent sterilization via bilateral tubal ligation was performed.

After delivery, the patient was able to be rapidly weaned from all IV medications and the chemical sympathectomy catheter. She was discharged home on postoperative day 4 asymptomatic and on the following oral medications: alpraz-olam 1 mg every 6 hours as needed for anxiety, metoprolol tartrate 12.5 mg twice daily, isosorbide mononitrate 30 mg daily, magnesium oxide 400 mg daily, nitroglycerin 0.4 mg sublingual as needed for chest pain, oxycodone-acetaminophen 10/325 every 6 hours as needed for severe pain, sertraline 50 mg daily, and verapamil 80 mg 3 times daily. Her baby did not survive. The infant died in the neonatal intensive care unit more than a month later.

## DISCUSSION

Our case demonstrates the difficulty in treating arrhythmia-inducing refractory vasospastic angina. This case was unique, with vasospasm documented in multiple coronary arteries and leading to unstable ventricular arrhythmia. The progression of symptoms throughout pregnancy required a prolonged stay in the intensive care unit and was dangerous for both the mother and fetus.

Pregnancy appears to be a trigger for coronary vasospasm, perhaps mediated by hormonal and nervous system changes throughout gestation. Pregnancy involves complex physiologic changes; primary systemic vasodilation leads to increases in sympathetic hormones and to the release of vasopressin and stimulation of the renal-angiotensin-aldosterone system,^8^ which may play a role in coronary vasospasm.

In our patient, high-dose IV medications and sedation did not control her vasospasm and subsequent ventricular arrhythmia. Chemical sympathectomy, which has been successful in refractory ventricular tachyarrhythmia,^9^ was temporizing but ultimately unable to completely suppress her symptoms. After having 7 shocks in the 5 years leading up to pregnancy, our patient had more than 20 shocks in a 6-week period during pregnancy despite intensive therapy. After considering the risks and benefits, the patient decided to proceed with tubal ligation to prevent future pregnancy.

## CONCLUSION

To our knowledge, this case is the first report of recurrent, drug-refractory vasospastic angina triggered by pregnancy. The physiologic changes of pregnancy appeared to be the trigger, making adequate treatment extremely challenging. The case highlights the aggressive therapy required to treat coronary vasospasm in pregnancy. A multidisciplinary approach was necessary with input from cardiology, maternal-fetal medicine, obstetrics, thoracic surgery, anesthesiology, and psychiatry. However, our patient had previously documented unstable tachyarrhythmia from coronary vasospasm. It is unknown if patients with controlled coronary vasospasm without arrhythmia could progress during pregnancy or if they could safely carry a pregnancy to term with conservative therapy.

## Notes

This article meets the Accreditation Council for Graduate Medical Education and the American Board of Medical Specialties Maintenance of Certification competencies for Patient Care and Medical Knowledge.
